# Circular RNA VANGL1 Facilitates Migration and Invasion of Papillary Thyroid Cancer by Modulating the miR-194/ZEB1/EMT Axis

**DOI:** 10.1155/2022/4818651

**Published:** 2022-03-08

**Authors:** Yangfeng Xiang, Wendong Wang, Jialei Gu, Jinbiao Shang

**Affiliations:** Department of Head and Neck Surgery, The Cancer Hospital of the University of Chinese Academy of Sciences (Zhejiang Cancer Hospital), Institute of Basic Medicine and Cancer (IBMC), Chinese Academy of Sciences, Hangzhou, Zhejiang 310022, China

## Abstract

Circular RNAs (circRNAs) are often aberrantly expressed in human tumors and also serve a critical regulatory role in papillary thyroid cancer (PTC). The aim of this study is to investigate the expression pattern and biological role of circVANGL1 in PTC. The results revealed that circVANGL1 was significantly upregulated in human PTC samples. In addition, high circVANGL1 expression was closely associated with adverse clinical parameters of PTC patients. Our in vitro experiments further indicated that the knockdown of circVANGL1 using siRNA obviously repressed migration, proliferation, EMT, and invasion of PTC cells, while opposite effects were induced by its overexpression. We further noted that circVANGL1 could interact with miR-194 directly in PTC, and serve as a ceRNA to regulate ZEB1 function. Moreover, miR-194 inhibition markedly abrogated the effects of circVANGL1 knockdown in PTC cells. Therefore, our results provide convincing evidence that circVANGL1 may exert oncogenic effects in PTC, partly via regulating the miR-194/ZEB1 axis.

## 1. Introduction

Thyroid cancer is the most common endocrine malignancy. Papillary thyroid cancer (PTC) constitutes nearly 80% of thyroid cancer cases, and its incidence has been on the rise in recent years [1]. PTC generally has a favorable prognosis, but due to invasiveness and metastasis, PTC can even become life-threatening [2]. Patients with advanced PTC only have a 5-year survival rate of 59% [3]. Consequently, it is of paramount importance to discover more valuable targets for PTC diagnosis and treatment.

Circular RNAs (circRNAs) are a novel type of RNA that, unlike linear RNAs, form a covalently closed loop structure without a 5′ cap or a 3′ poly-A tail [4]. They are generated from “direct back-splicing” and exon “skipping” of pre-mRNA transcripts [5]. Many evidence demonstrates that circRNAs are frequently implicated in the regulation of diverse pathophysiological processes, including cell proliferation, apoptosis, metastasis, and cancer progression [6]. circRNA VANGL1 (circVANGL1), derived from two exons of the Van Gogh-like 1 (VANGL1) gene, was previously identified as an oncogene in human bladder cancer [7–9]. This work was carried out to reveal the expression pattern and biological role of circVANGL1 in PTC.

## 2. Materials and Methods

### 2.1. Human Tissue Samples

77 pairs of PTC tissues and adjacent nontumor tissues of patients were collected during surgical resection. All participants did not receive any preoperative therapy. All the procedures involving human tissues got approval from the ethics committee of the hospital. All subjects signed informed consent.

### 2.2. Cell Culture and Transfection

RPMI-1640 Medium (Invitrogen, Carlsbad, CA, USA) containing 10% FBS was adopted for the culture of a normal thyroid epithelial cell line (Nthy-ori 3-1) and three human PTC cell lines (K-1, TPC-1, and IHH-4).

To obtain the circVANGL1 overexpression plasmid, circVANGL1 cDNA was cloned into a pLVX-cir vector (Genomeditech Co., Ltd., Shanghai, China). siRNA targeting circVANGL1 (si-circVANGL1), negative control (NC) siRNA (si-NC), miR-194 mimics, miR-194 inhibitor, NC mimics, and NC inhibitor were obtained commercially. Lipofectamine 2000 (Invitrogen) was employed for cell transfection.

### 2.3. RT-qPCR Analysis

After extraction, the PrimeScript RT reagent Kit (TaKaRa, Dalian, China) was employed to obtain cDNA through reverse transcription of RNA. Thereafter, the SYBR Green PCR Kit (TaKaRa) was adopted for the reaction. Quantitative analysis of relative gene expression was implemented by the 2^−ΔΔCt^ method [10], with GAPDH or U6 as an internal control.

### 2.4. CCK-8 Assay

Cell counting kit-8 (CCK-8) (Beyotime, Shanghai, China) was employed for cell proliferation. 96-well plates were seeded with cells (2 × 10^3^ cells/well) and cultured for 24–96 h. Each well was added with 10 *μ*L of CCK-8 reagent and mixed for additional 2 h. Absorbance at 450 nm was then measured by using a microplate reader (Tecan, Salzburg, Austria).

### 2.5. Transwell Assay

The upper chamber of transwell plates (8 *μ*m pore size) was added with 2 × 10^4^ cells suspended in medium without serum. The lower chambers were added with medium containing 10% FBS. Two days later, the cells that had moved to the lower chambers were fixed with 4% paraformaldehyde, stained with 0.1% crystal violet, and observed under a light microscope.

### 2.6. Western Blot Analysis

Proteins with identical quantities were separated using SDS-PAGE and then transferred onto PVDF membranes. After blocking with 5% BSA, the membranes were incubated with specific primary antibodies against E-cadherin (dilution, 1 : 2,000; Abcam, Cambridge, UK), N-cadherin (1 : 2,000; Abcam), vimentin (1 : 2,000; Abcam), ZEB1 (1 : 2,000; Abcam), or GAPDH (1 : 2,000; Abcam) at 4°C overnight, followed by incubation with HRP-conjugated secondary antibody (1 : 10,000; Santa Cruz Biotechnology, Inc., Dallas, TX, USA) at room temperature for 2 h. Using an enhanced chemiluminescence kit (Beyotime), the bands were visualized. GAPDH served as a protein-loading control.

### 2.7. Dual-Luciferase Reporter Assay

The fragment of circVANGL1 or ZEB1 mRNA with predicted miR-194-binding sites was inserted into the pmiR-GLO dual-luciferase miRNA target expression vector (Promega, Madison, WI, USA). Cells were cotransfected with the luciferase reporters and miR-194 mimics or NC mimics using Lipofectamine 2000. Two days later, the luciferase activity was detected using the Dual-Luciferase Reporter Assay System (Promega).

### 2.8. Statistical Methods

GraphPad Prism 6.0 and SPSS 18.0 were employed for data analysis. Differences within groups were assessed by Student's *t*-test, *χ*^2^ test, or one-way ANOVA, as appropriate. *P* < 0.05 was deemed as statistically considerable.

## 3. Results

### 3.1. circVANGL1 Is Overexpressed in PTC

**Figure 1:**
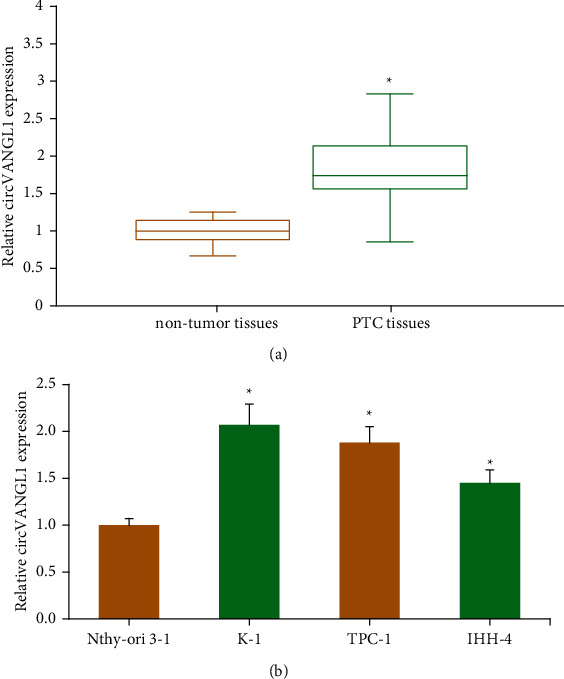
circVANGL1 is overexpressed in PTC. (a) circVANGL1 level in PTC tissues and matched nontumor tissues. (b) circVANGL1 level in PTC cell lines and Nthy-ori 3-1 cells. ^*∗*^*P* < 0.05 vs. nontumor tissues or Nthy-ori 3-1 cells.

First, Figure 1(a) showed that, compared to adjacent nontumor tissues, the circVANGL1 level was notably increased in PTC tissues. As illustrated in Figure 1(b), circVANGL1 level was also remarkably increased in a panel of PTC cell lines versus Nthy-ori 3-1 cells.

Next, according to the mean circVANGL1 expression, these PTC patients were allocated into two groups.  Table 1 manifested that high intratumoral circVANGL1 expression was markedly correlated with adverse clinical parameters of PTC patients, including lymph node metastasis (*P*=0.017) and advanced TNM stage (*P*=0.007).

### 3.2. circVANGL1 Promotes PTC Cell Proliferation and Invasion

**Figure 2:**
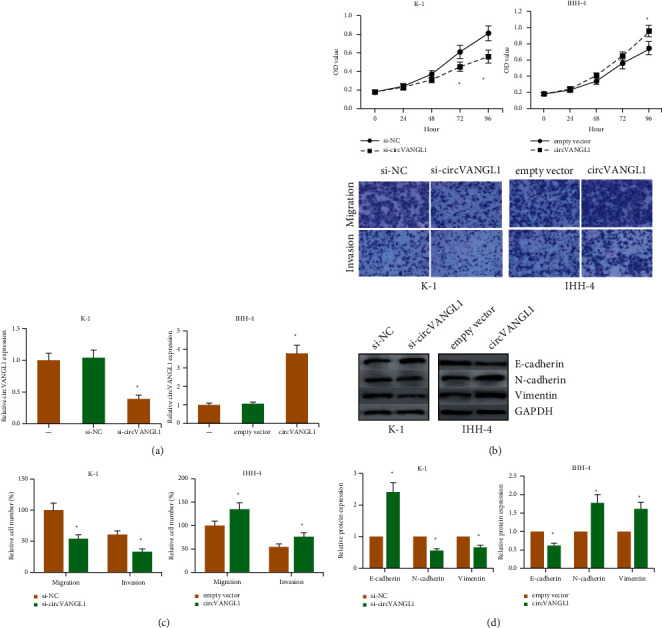
circVANGL1 promotes PTC cell proliferation and invasion. (a) circVANGL1 level in PTC cells after transfection. (b) Proliferation of PTC cells after circVANGL1 overexpression/knockdown. (c) PTC cell migration and invasion after circVANGL1 overexpression/knockdown. (d) EMT-related protein level in PTC cells after circVANGL1 overexpression/knockdown. ^*∗*^*P* < 0.05 vs. si-NC or empty vector group.

Loss and gain-of-function assays were then designed and conducted. We overexpressed circVANGL1 in IHH-4 cells and knocked down circVANGL1 in K-1 cells. The overexpression and knockdown efficiencies were confirmed by RT-qPCR analysis (Figure 2(a)). As exhibited in Figure 2(b), the proliferation of K-1 cells was markedly suppressed by circVANGL1 knockdown, while circVANGL1 overexpression notably enhanced the proliferation of IHH-4 cells. Based on the transwell assay, we uncovered that after circVANGL1 knockdown, the invasion and migration of K-1 cells were notably repressed, while these behaviors were markedly enhanced by circVANGL1 overexpression in IHH-4 cells (Figure 2(c)). Furthermore, western blot analysis indicated that after circVANGL1 knockdown, E-cadherin levels were increased, while N-cadherin and vimentin were markedly reduced in K-1 cells (Figure 2(d)). However, circVANGL1 overexpression led to the opposite effects in IHH-4 cells.

### 3.3. circVANGL1 Functions as a ceRNA for miR-194 in PTC

**Figure 3:**
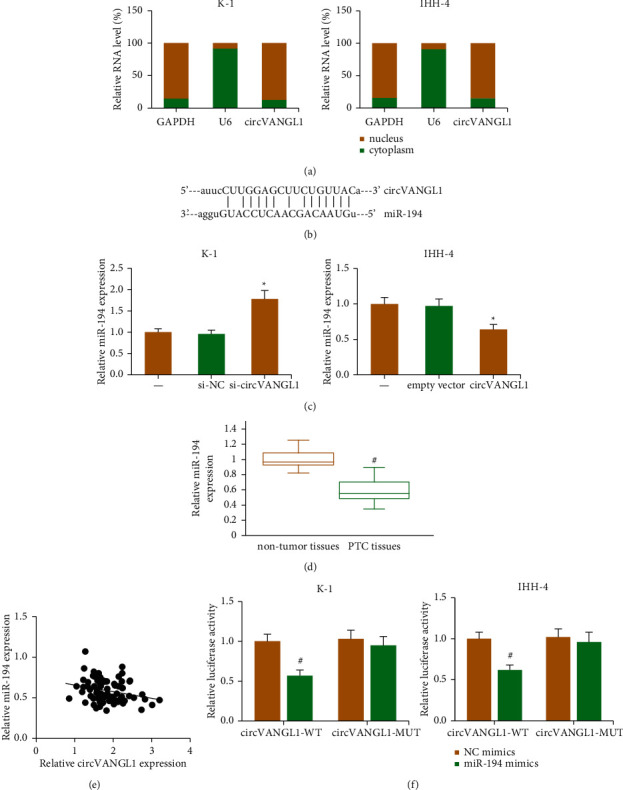
circVANGL1 functions as a ceRNA for miR-194 in PTC. (a) Subcellular location of circVANGL1 in PTC cells. (b) Putative targeting sites for miR-194 on circVANGL1 fragment. (c) miR-194 level in PTC cells after circVANGL1 overexpression/knockdown. (d) miR-194 level in PTC tissues and matched nontumor tissues. (e) Pearson correlation of circVANGL1 and miR-194 level in PTC tissues. (f) Luciferase activities of reporters in PTC cells after cotransfection with miR-194 mimics/NC mimics. ^*∗*^*P* < 0.05 vs. si-NC or empty vector group; ^#^*P* < 0.05 vs. NC mimics group or nontumor tissues.

The mechanisms underlying the functional role of circVANGL1 in PTC were further explored. As demonstrated in Figure 3(a), circVANGL1 was predominantly distributed in the cytoplasmic fractions of K-1 and IHH-4 cells, indicating it may function as a ceRNA. We then used the starBase online software to predict the potential target miRNA of circVANGL1 and discovered the complementary sequences between miR-194 and circVANGL1 fragment (Figure 3(b)). In addition, circVANGL1 knockdown notably increased miR-194 expression in K-1 cells, while its expression was decreased by circVANGL1 overexpression in IHH-4 cells (Figure 3(c)). In PTC tissues, miR-194 expression was markedly reduced (Figure 3(d)), and its expression was negatively associated with circVANGL1 expression (*P*=0.031; Figure 3(e)). Moreover, miR-194 mimics dramatically suppressed the luciferase activity of vector containing circVANGL1-WT in K-1 and IHH-4 cells (Figure 3(f)).

### 3.4. ZEB1 Is a Target of miR-194 in PTC

**Figure 4:**
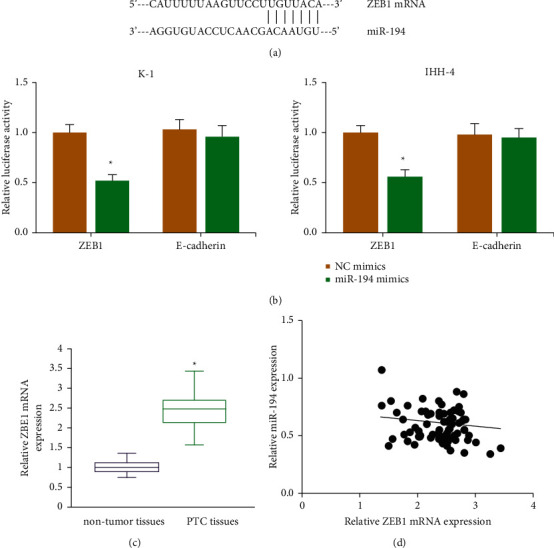
ZEB1 is a target of miR-194 in PTC. (a) Putative targeting sites for miR-194 on ZEB1 mRNA fragment. (b) Luciferase activities of reporters in PTC cells after cotransfection with miR-194 mimics/NC mimics. (c) ZEB1 mRNA level in PTC tissues and matched nontumor tissues. ^*∗*^*P* < 0.05 vs. NC mimics group or nontumor tissues. (d) Pearson correlation analysis of miR-194 and ZEB1 mRNA expression in PTC tissues.

ZEB1 mRNA fragment may contain the binding sites of miR-194 (Figure 4(a)), as predicted by TargetScan database. miR-194 mimics strikingly suppressed the luciferase activity of vector containing ZEB1-WT in K-1 and IHH-4 cells (Figure 4(b)). We further found that with a comparison to adjacent nontumor tissues, ZEB1 mRNA expression was markedly increased in PTC tissues (Figure 4(c)), and its level was also negatively correlated with miR-194 expression (*P*=0.033; Figure 4(d)).

### 3.5. miR-194 Inhibition Blocks the Role of circVANGL1 Knockdown in PTC Cells

**Figure 5:**
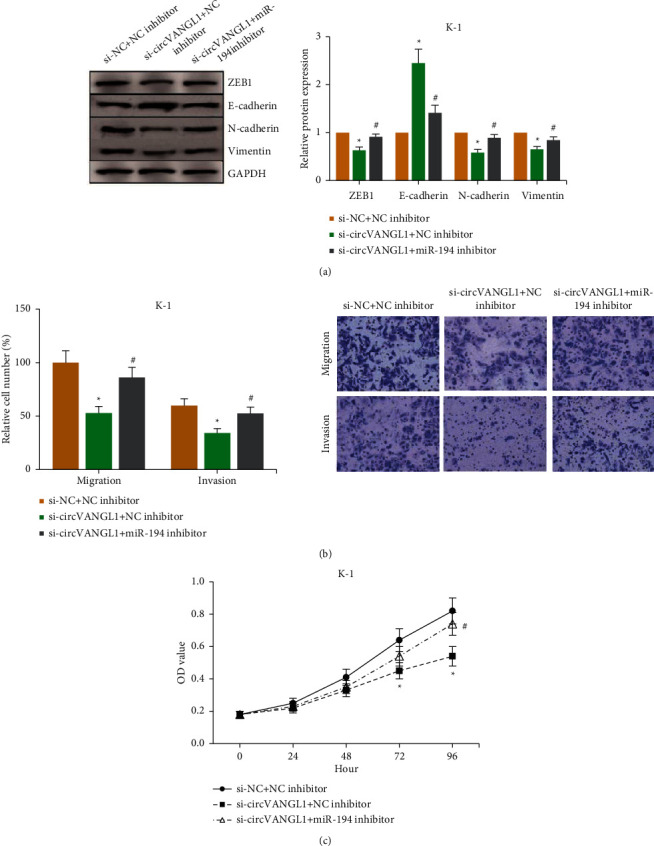
miR-194 inhibition blocks the effects of circVANGL1 knockdown in PTC cells. (a) EMT-related protein level in PTC cells after miR-194 inhibition. (b) PTC cells' migration and invasion after miR-194 inhibition. (c) Proliferation of PTC cells after miR-194 inhibition. ^*∗*^*P* < 0.05 vs. si-NC + NC inhibitor group; ^#^*P* < 0.05 vs. si-circVANGL1 + NC inhibitor group.

We then inhibited miR-194 expression in K-1 cells with circVANGL1 knockdown, and found that the reduced ZEB1 protein level was markedly rescued, accompanied by the promotion of EMT (Figure 5(a)). miR-194 inhibition also obviously counteracted the effects of circVANGL1 knockdown on suppressing K-1 cell migration and invasion (Figure 5(b)). The CCK-8 assay confirmed that miR-194 inhibition diminished the inhibitory effect of circVANGL1 knockdown on proliferation of K-1 cells (Figure 5(c)).

## 4. Discussion

Tumor progression is a complex, multistage process involving many genetic and environmental factors. Recently, a lot of circRNAs have been identified, and their regulatory roles in tumor progression have been well documented [11]. They may also be putative biomarkers for the diagnosis and evaluation of PTC progression [12]. In this study, we analyzed the expression profile and functional role of circVANGL1 in PTC.

Our results showed that circVANGL1 was strikingly upregulated in human PTC samples, and its high level was strongly associated with adverse clinical parameters of PTC patients, suggesting a clinicopathological role of circVANGL1 in PTC. Tumor cell proliferation, migration, and invasion are representative indicators of malignant phenotypes, and through a series of functional experiments, we further noted that circVANGL1 knockdown remarkably inhibited these malignant behaviors of PTC cells, while its overexpression showed opposite effects. As a key step in cancer metastasis, EMT endows cancer cells with a more motile and invasive phenotype, and it is also implicated in the metastatic progression of PTC [13]. In melanoma cells, the knockdown of circVANGL1 inhibits TGF-*β*-induced EMT [14], and our results indicate that circVANGL1 can also promote EMT in PTC cells.

A growing body of evidence has suggested that circRNAs can function as competitive endogenous RNAs (ceRNAs), which compete for microRNAs (miRNAs) to regulate cancer progression [15]. In non-small-cell lung cancer, circVANGL1 serves as a ceRNA, becoming a sink for miR-195 [16]. CircVANGL1 also functions as a sponge for miR-145-5p in bladder cancer [9]. miR-194 is widely accepted as a tumor suppressor in several human cancers [17–19], and this research revealed that its expression was also markedly decreased in PTC samples. Furthermore, we uncovered that circVANGL1 could bind to miR-194 directly and relieve suppression of the target, ZEB1, in PTC. Further rescue assays demonstrated that miR-194 inhibition could abolish the inhibitory role of circVANGL1 knockdown on the malignant behaviors of PTC cells.

In conclusion, the findings of this paper suggested that circVANGL1 is highly expressed in PTC samples and promotes PTC progression partly by modulating the miR-194/ZEB1/EMT axis. Therefore, circVANGL1 may be exploited as a potential therapeutic target for PTC patients.

## Figures and Tables

**Table 1:** Correlation between circVANGL1 expression and clinical pathological characteristics in PTC.

Characteristics	Total number (*N* = 77)	circVANGL1 expression		*P* value
		Low (*N* = 44)	High (*N* = 33)	
Age (years)				0.319
<45	33	21	12	
≥45	44	23	21	
Gender				0.666
Male	23	14	9	
Female	54	30	24	
Tumor size (cm)				0.106
<2	52	33	19	
≥2	25	11	14	
Lymph node metastasis				0.017
No	49	33	16	
Yes	28	11	17	
TNM stage				0.007
I–II	46	32	14	
III–IV	31	12	19	

## Data Availability

The data used to support the findings of this study are available from the corresponding author upon request.
